# *cis*-Urocanic Acid Attenuates Acute Dextran Sodium Sulphate-Induced Intestinal Inflammation

**DOI:** 10.1371/journal.pone.0013676

**Published:** 2010-10-27

**Authors:** Eric Albert, John Walker, Aducio Thiesen, Thomas Churchill, Karen Madsen

**Affiliations:** 1 Department of Medicine, University of Alberta, Edmonton, Alberta, Canada; 2 Department of Laboratory Medicine and Pathology, University of Alberta, Edmonton, Alberta, Canada; 3 Department of Surgery, University of Alberta, Edmonton, Alberta, Canada; New York University, United States of America

## Abstract

On exposure to sunlight, urocanic acid (UCA) in the skin is converted from *trans* to the *cis* form and distributed systemically where it confers systemic immunosuppression. The aim of this study was to determine if administration of *cis-*UCA would be effective in attenuating colitis and the possible role of IL-10. Colitis was induced in 129/SvEv mice by administering 5% dextran sodium sulfate (DSS) for 7 days in drinking water. During this period mice received daily subcutaneously injections of *cis-*UCA or vehicle. To examine a role for IL-10, 129/SvEv IL-10^−/−^ mice were injected for 24 days with *cis-*UCA or vehicle. Clinical disease was assessed by measurement of body weight, stool consistency, and presence of blood. At sacrifice, colonic tissue was collected for histology and measurement of myeloperoxidase and cytokines. Splenocytes were analyzed for CD4+CD25+FoxP3+ T-regulatory cells via flow cytometry. Murine bone-marrow derived antigen-presenting cells were treated with lipopolysaccharide (LPS) ± UCA and cytokine secretion measured. Our results demonstrated that *cis-*UCA at a dose of 50 µg was effective in ameliorating DSS-induced colitis as evidenced by reduced weight loss and attenuated changes in colon weight/length. This protection was associated with reduced colonic expression of CXCL1, an increased expression of IL-17A and a significant preservation of splenic CD4+CD25+FoxP3+ T-regulatory cells. *cis-*UCA decreased LPS induced CXCL1, but not TNFα secretion, from antigen-presenting cells *in vitro*. UCA reduced colonic levels of IFNγ in IL-10^−/−^ mice but did not attenuate colitis. In conclusion, this study demonstrates that *cis*-urocanic acid is effective in reducing the severity of colitis in a chemically-induced mouse model, indicating that pathways induced by ultraviolet radiation to the skin can influence distal sites of inflammation. This provides further evidence for a possible role for sunlight exposure in modulating inflammatory disorders.

## Introduction

Inflammatory bowel diseases (IBD), consisting of Crohn's disease (CD) and ulcerative colitis (UC), are chronic inflammatory conditions of the gut believed to occur in genetically predisposed individuals who are exposed to unknown environmental and microbial triggers [1], [2], [3], [4]. Epidemiological evidence suggests that ultraviolet radiation may play a protective role in several autoimmune disorders, including multiple sclerosis, rheumatoid arthritis, and inflammatory bowel disease [5]. The geographical distribution of these diseases, including IBD, inversely correlates with exposure to ultraviolet radiation. There is a North-South gradient in IBD incidence in Europe and North America, where both CD and UC increase in concurrence with latitude increases [6], [7], [8].

One well studied mechanism by which sunlight exposure conveys beneficial effects involves the synthesis of vitamin D in the skin, which has been subsequently shown to have immunomodulatory properties by downregulating Th1 driven immune responses[9]. Another mechanism by which sunlight conveys immunomodulatory properties was reported by De Fabo and Noonan when they generated an action spectrum to determine how the transduction of UV radiation into a biochemical signal occurs [10]. It was determined that narrow-band UV radiation between the wavelengths of 250 and 320 nm photoisomerizes a photoreceptor known as *trans-*urocanic acid into *cis-*urocanic acid in the stratum corneum of the epidermis[10]. Several other groups have reported that upon photoisomerization, *cis*-urocanic acid (*cis-*UCA) is distributed systemically, where it has been shown to convey both local and systemic immunosuppression [11]. *cis-*UCA has shown effectiveness in down-regulating hypersensitivity reactions, decreasing the presentation of tumor antigen by Langerhans cells, and suppressing cell mediated immunity[12], [13], [14]. There are numerous pathways in which *cis-*UCA can mediate effects on the immune system as well as multiple cellular targets including neutrophils, monocytes, keratinocytes, T-cells and epithelial cells[11], [15], [16], [17]. *cis-*UCA has been shown to increase IL-10 secretion by CD4+ T-cells *in vitro* and ultraviolet-mediated tolerance and suppression of contact hypersensitivity and delayed-type hypersensitivity have been shown to be mediated by IL-10 secreting T-regulatory cells [18], [19], [20]. In primary human keratinocytes, *cis-*UCA has been shown to upregulate expression of prostaglandin-endoperoxide synthase-2 and cause increased secretion of PGE2; this can potently activate NF-κB resulting in the secretion of pro-inflammatory cytokines such as TNFα, IL-6 and IL-8 [21]. Together, this data indicates that *cis-*UCA is a pleiotropic molecule with numerous systemic effects.

The aim of this study was to determine if administration of *cis-*UCA subcutaneously would suppress inflammation in a distal site such as the colon. We hypothesized that the immunomodulatory effects of *cis-*UCA would result in an attenuation of colitis due to its documented ability to decrease inflammatory responses in the skin. In concordance with this hypothesis, our findings demonstrate that *cis-*UCA, administered via the skin, is capable of modulating inflammatory responses in the colon during chemically-induced colitis resulting in attenuated colonic inflammation.

## Materials and Methods

### Animals and induction of colitis

For all animal experiments 8–12 week old homozygous IL-10 gene-deficient mice (IL-10^−/−^), generated on a 129 Sv/Ev background and wild-type 129 Sv/Ev controls were used. Animals were housed under specific pathogen-free conditions and allowed free access to regular water and food. In wild-type mice, acute colitis was induced by the administration of dextran sodium sulfate (DSS) (M.W. = 36,000–50,000, MP Biomedicals) at a concentration of 5% weight per volume in regular drinking water for 7 days. Daily weights, the presence or absence of blood, and stool consistencies were recorded. In DSS experiments mice were injected daily subcutaneously along the dorsal axis with either 5 or 50 µg *cis-*UCA or vehicle (PBS). IL-10^−/−^ mice develop a chronic colitis over several weeks following weaning that is characterized by a patchy transmural inflammation limited to the colon [22]. In order to determine if *cis*-UCA would attenuate the development of colitis in this model, IL-10^−/−^ mice were injected every second day for 24 days subcutaneously along the dorsal axis with either 5 or 50 µg *cis-*UCA or vehicle control (PBS). All animals were sacrificed via cervical dislocation, and the entire large intestine and spleens were removed. DSS experiments were repeated three times with 4–6 mice per group. *cis-*UCA treatment of IL-10−/− mice was carried out in groups of 5–6 mice. All experiments were approved by the University of Alberta Animal Care and Use committee for health sciences according to Protocol #138.

### *Cis-*urocanic acid

Commercially available *cis*-urocanic acid (Sigma Chemical, St. Louis, MO) was dissolved at a concentration of 1 mg/ml in sterile PBS. All aliquots were stored in the dark to ensure their stability.

### Histological injury and disease activity grading

The large intestine was removed free of fatty tissue and mesenteric lymph nodes. Entire colon length was measured from the proximal colon to the rectum. The entire colon was flushed with ice cold PBS containing penicillin/streptomycin and gentamycin. Two colonic segments were frozen at −20°C and saved for sonication to analyze cytokine and myeloperoxidase levels. A third segment was fixed in 10% neutral buffered formalin and paraffin-embedded. Paraffin embedded tissue sections were cut 5 µm thick and stained with Hematoxylin and Eosin (H&E) for general histology. Disease activity scores for DSS treated mice were based on the cumulative scores (0–10) from 3 different parameters: Stool consistency (0 = normal, 1 = soft but still formed, 2 = very soft, 3 = diarrhea), presence of blood in stool (0 = negative heamocccult, 1 = positive hemoccult, 2 = blood traces visible in stool, 3 = rectal bleeding) and histological changes (0–4). Histological scoring was performed by two different pathologists in a blinded manner. Criteria for histological scoring of DSS treated mice was based on the following parameters - no evidence of inflammation (score = 0); low level of inflammation with scattered infiltrating mononuclear cells and neutrophils (score = 1); moderate inflammation with multiple foci of neutrophils (score = 2); high degree of inflammation with increased vascular density and marked wall thickening (score = 3); maximal severity of inflammation with transmural leukocyte infiltration and loss of goblet cells accompanied by ulceration (score = 4). Histological scoring of intestinal inflammation for IL-10−/− mice was performed using the scheme previously defined by Madsen et al. [22]. Briefly, histological grades (ranging from 0–10) represent the sum of four scoring criteria: mucosal ulceration (0–3), epithelial hyperplasia (0–3), lamina propria mononuclear infiltration (0–2), and lamina propria neutrophilic infiltration (0–2).

### Cytokine and myeloperoxidase measurement

At sacrifice, a section of colon was removed, weighed, and immediately flash-frozen. Samples were kept at −70 C until analysis by ELISA. The following DuoSet® ELISA Development System antibody sets from R&D Systems (Minneapolis, MN) were used: mouse IL-10 (DY417), mouse IL-6 (DY406), mouse IFN-γ (DY485), mouse TNFα (DY410), mouse CXCL1/KC (DY453), mouse IL-23 (DY1887), and human CXCL8/IL-8 (DY208). Mouse IL-17A was measured using the ELISA Ready-Set-Go antibody kit from eBioscience (cat#88-7371). Levels of myeloperoxidase (MPO) in colonic tissue were measured using an ELISA kit (Cell Sciences, Canton, Massachusetts, USA).

### Preparation and activation of murine spleen cells

Spleens were removed aseptically from mice and teased into single cell suspensions in IMag buffer (BD Biosciences) in sterile Petri dishes on ice. The cell suspension was centrifuged at 200 g for 10 min and the cell pellet resuspended in lysis medium (1 volume of 0.17 M Tris, pH 7.6, 9 volumes of 0.16 M NH4Cl) to remove red blood cells and repelleted. Supernatants were aspirated and pellets resuspended in 1 ml IMag buffer. Cell numbers were determined by coulter counter. 1×10^6^ total splenocytes were stained for CD4, CD25, FoxP3 and IL-10 and analyzed via flow cytometry. Cells positive for CD4 and CD25 were gated and analyzed for their expression of FoxP3, IL-10, and IL-17A. Cell numbers are expressed as percentages of total cells. Dot plots and histograms were obtained using FCS express version 3 research edition.

### Generation of bone marrow-derived antigen-presenting cells (BM-APCs)

Bone marrow was harvested and pooled from 3 wild-type 129 Sv/Ev mice. Femurs of mice were removed, cleaned of all tissue and rinsed in 70% ethanol on ice for 2–5 min. Femurs were flushed vigorously with media containing RPMI 1640, 100 U/ml penicillin, 100 µg/ml streptomycin, 2 mM L-Glutamine, 50 µM mercaptoethanol, 10% heat inactivated FBS. Cells were seeded in 100 mm sterile Petri dishes at a density of 2×10^6^ cells in media containing 20 ng/ml rmGM-CSF (PeproTech, Rocky Hill, NJ) and 5 ng/ml rmIL-4 (PeproTech, Rocky Hill, NJ). Cells were maintained at 37°C in a 5% CO2 atmosphere. Cultures were re-fed after 3 days with fresh media. On days 6 and 8, half of the cell supernatant was collected, centrifuged at 1100 rpm for 5 minutes and resuspended in media containing rmGM-CSF and rmIL-4 and returned to the Petri dish. On day 10 cells were used for experiments. Experiments utilizing BM-APCs were carried out in triplicate using bone marrow pooled from 3 wild-type 129 Sv/Ev mice for each experiment. Cells were plated at a density of 1×10^6^ cells/ml in 24 well tissue culture plates (Falcon, NJ) with each treatment performed in triplicate over 3 separate experiments.

### Statistical analysis

Data are expressed as means ± SEM. Data were tested for normality of distribution and analyses performed using the statistical software SigmaStat (Jandel Corporation, San Rafael, CA). Differences between means were evaluated using analysis of variance or paired *t*-tests where appropriate. Specific differences were tested using the Student-Newman-Keuls test.

## Results

### *cis-*UCA attenuates acute DSS-induced colitis

**Figure 1:**
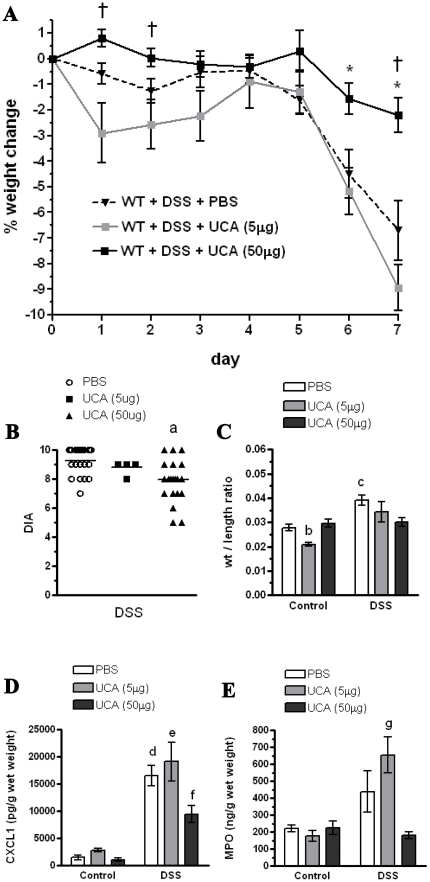
*cis*-UCA (50 µg) ameliorates dextran sodium sulfate (DSS)-induced disease in 129Sv/Ev mice. 129Sv/Ev mice were treated with 5% DSS for 7 days to induce acute colitis. Beginning on the first day of DSS administration mice were injected subcutaneously along the dorsal axis daily for 7 days with either 5 or 50 µg *cis*-UCA, or vehicle (PBS). Measurements were taken on Day 7. DSS treatment induced weight loss in mice, and this was attenuated by treatment with *cis*-UCA (50 µg). Mice receiving *cis-*UCA and DSS at the low dose (5 µg) had a transient increased weight loss compared with mice receiving the high dose of *cis-*UCA and DSS at days 1 and 2 (A). Total colonic disease activity score (DIA) was reduced by *cis*-UCA (50 µg) treatment (B). DSS treatment increased the colonic weight/length ratio, and this was prevented by treatment with *cis*-UCA (50 µg). Mice receiving the low dose of *cis-*UCA had a reduced weight/length ratio compared with PBS-treated and *cis-*UCA (50 µg) mice (C). DSS treatment increased levels of the chemokine, CXCL1, in colonic tissue, and this was attenuated by *cis*-UCA (50 µg) treatment (D). Total tissue levels of myeloperoxidase (MPO) were significantly increased in *cis-*UCA (5 µg) + DSS treated mice (E). n = 5–25 mice for all measurements. †: p<0.05 UCA (50 µg) compared with UCA (5 µg); *: p<0.05 UCA (50 µg) compared with PBS. a: p<0.01 UCA (50 µg) compared with PBS; b: p<0.01 Control UCA (5 µg) compared with control PBS, control UCA (50 µg), PBS+DSS, DSS+UCA (5 µg), and DSS+UCA (50 µg). c: p<0.05 PBS+DSS compared with control PBS, control UCA (5 µg), control UCA (50 µg), and DSS+UCA (50 µg). d: p<0.01 DSS+PBS compared with control PBS, control UCA (5 µg), control UCA (50 µg), and DSS+UCA (50 µg). e: p<0.01 DSS+UCA (5 µg) compared with control PBS, control UCA (5 µg), control UCA (50 µg), and DSS+UCA (50 µg). f: p<0.05 DSS+UCA(50 µg) compared with control PBS and control UCA (50 µg). g: p<0.05 DSS+UCA (5 µg) compared to control UCA (5 µg) and DSS+UCA (50 µg).

Following 7 days of DSS administration, mice receiving the high dose of *cis-*UCA (50 µg) lost significantly less weight compared with those mice receiving vehicle and the low dose of *cis-*UCA (5 µg) (Fig. 1A). In addition, mice receiving the high dose of *cis-*UCA had lower overall disease activity score compared to values seen in control mice (Fig. 1B). Following 7 days of DSS administration, mice receiving *cis-*UCA (50 µg) had a stool consistency score of 2.2±0.7 and a blood score of 2.0±0.87 (N = 22) while control mice receiving PBS had a stool consistency score of 2.8±0.4 and a blood score of 2.7±0.4 (N = 25). Mice receiving high dose *cis-*UCA (50 µg) also maintained colonic weight to length ratios to values seen in control mice (Fig. 1C). These values, combined with the weight change score, resulted in a significant attenuation of disease score in the mice treated with *cis-*UCA at the high dose of 50 µg (Fig. 1B). Interestingly, in the absence of DSS, mice administered the low dose of *cis-*UCA (5 µg) had a decreased colon weight to length ratio compared to both PBS control and the high dose *cis-*UCA (50 µg) (Fig. 1C). DSS treatment resulted in increased colonic levels of the neutrophil chemokine, CXCL1 (Fig. 1D) Mice receiving the high dose of *cis-*UCA had reduced levels of CXCL1 (Fig. 1D). Mice receiving the low dose of *cis-*UCA had similar levels of MPO (Fig. 1E) and CXCL1 to the PBS-treated DSS group.

### Effect of *cis-*UCA on colonic cytokines

**Figure 2:**
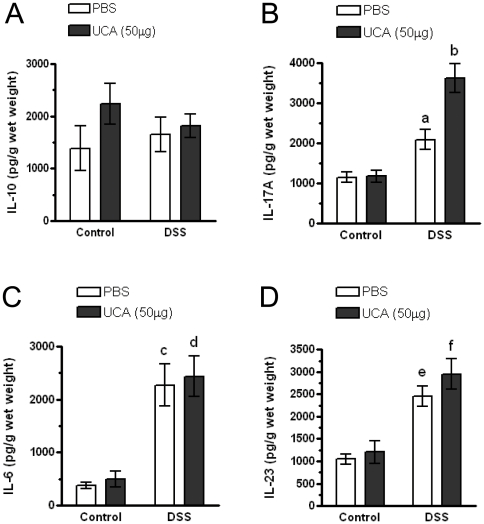
Effect of *cis*-UCA and DSS on tissue cytokines. 129Sv/Ev mice were treated with 5% DSS for 7 days to induce acute colitis. Beginning on the first day of DSS administration mice were injected subcutaneously along the dorsal axis daily for 7 days with 50 µg *cis*-UCA or vehicle (PBS). Colonic tissue was ultrasonicated and supernatants were analyzed for total levels of cytokines. *cis*-UCA and DSS had no significant effects on tissue levels of IL-10 (A). DSS treated mice had increased levels of IL-17, and these were further increased in mice treated with *cis*-UCA (50 µg) (B). DSS treated mice had increased levels of colonic IL-6 (C) and IL-23 (D) and these values were not altered by *cis*-UCA (50 µg) n = 5–25 for all measurements. a: p<0.01 DSS+PBS compared with control PBS, control UCA (50 µg), and DSS+UCA (50 µg). b: p<0.01 DSS+UCA (50 µg) compared with control PBS and control UCA (50 µg). c: p<0.01 DSS+PBS compared with control PBS, and control UCA (50 µg). d: p<0.01 DSS+UCA (50 µg) compared with control PBS, and control UCA (50 µg). e: p<0.01 DSS+PBS compared with control PBS, and control UCA (50 µg). f: p<0.01 DSS+UCA (50 µg) compared with control PBS, and control UCA (50 µg).

In the skin *cis-*UCA has been shown to modulate various cytokine responses including IL-10, TNFα, IL-6 and IL-8 secretion [18], [19], [20], [21]. However, whether administration of *cis-*UCA via the skin has effects in the gut has not been determined. We examined levels of IL-6, IL-10, IL-17A, and IL-23 in the colons following 7 days of *cis-*UCA treatment, and also following the induction of colitis. Treatment of mice with *cis-*UCA in the absence of DSS resulted in no significant change in levels of colonic IL-10 (Fig. 2A), IL-17A (Fig. 2B), IL-6 (Fig. 2C), and IL-23 (Fig. 2D). DSS treatment resulted in a significant increase in levels of IL-17A, IL-6, and IL-23 in both *cis-*UCA and PBS treated groups. Interestingly, in the DSS-treated group, mice receiving the high dose of *cis-*UCA had significantly elevated IL-17A levels compared to mice receiving DSS and PBS (Fig. 2B).

### *cis-*UCA effects on splenocyte TH17 and T regulatory cells

**Figure 3:**
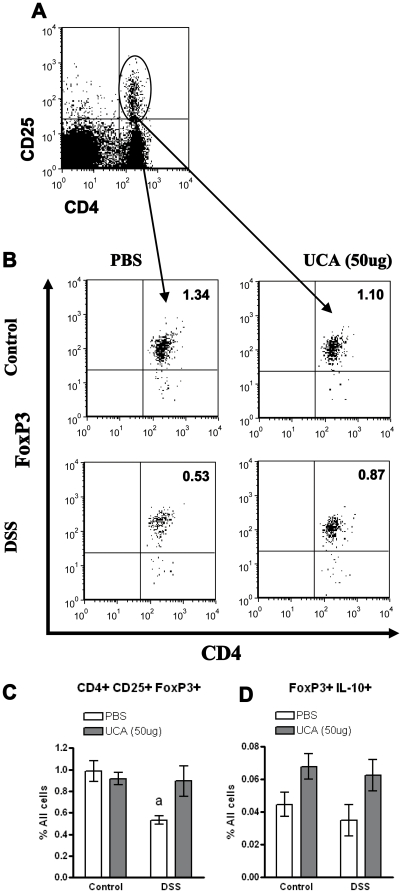
Treatment with *cis*-UCA (50 µg) maintains splenic T-regulatory cell populations. 129Sv/Ev mice were given 5% DSS for 7 days and treated with *cis*-UCA (50 µg) or PBS vehicle daily. Spleens from mice were removed on day 7. Total splenocytes were stained for CD4, CD25, FoxP3 and IL-10 and analyzed via flow cytometry. Cells positive for CD4 and CD25 (A) were gated and analyzed for their expression of FoxP3 (B). DSS treated mice had reduced levels of CD4+CD25+FoxP3+ cells when cell numbers were expressed as percentage of total cells (C). cis-UCA (50 µg) treated mice had similar numbers of CD4+CD25+FoxP3+ cells as compared with control mice (D). DSS and *cis*-UCA (50 µg) treatment did not alter levels of IL-10 secreting CD4+CD25+FoxP3+ cells (D). n = 4–13 for all measurements. a; p<0.05 DSS+PBS compared to control PBS, control UCA, and DSS+UCA (50 µg).

IL-17A is secreted by numerous cell types, including Th17 cells, γδ T cells, NK cells, and neutrophils, and has an important role in host defence [23]. Due to the increased levels of IL-17A seen in the colon, we assessed splenocytes to determine if *cis-*UCA treatment increased the number of Th17 cells. However, there was no difference in the amount of Th17 cells in the spleen in any of the groups (data not shown). In that a balance exists between Th17 cells and the induced FoxP3+ subset of regulatory CD4+CD25+ T cells, we also examined whether treatment with *cis-*UCA altered CD4+CD25+FoxP3+ T-cells in the spleen. Treatment of mice with *cis-*UCA (50 ug) did not result in any change in the percentage of CD4+CD25+FoxP3+ cells as compared with the control group (Fig. 3C). During acute DSS-colitis, mice receiving vehicle showed a significant drop in total percentage of CD4+CD25+FoxP3+ splenocytes, while mice receiving *cis-*UCA maintained pre-DSS levels of CD4+CD25+FoxP3+ cells and were significantly higher than PBS control levels (Fig. 3C). There was a similar trend towards increased levels of FoxP3+IL-10+ cells in the DSS-UCA treated group, however this did not reach significance (Fig. 3D). (p = 0.11)

### *In vitro* effects of cis-urocanic acid on bone marrow derived antigen-presenting cells

**Figure 4:**
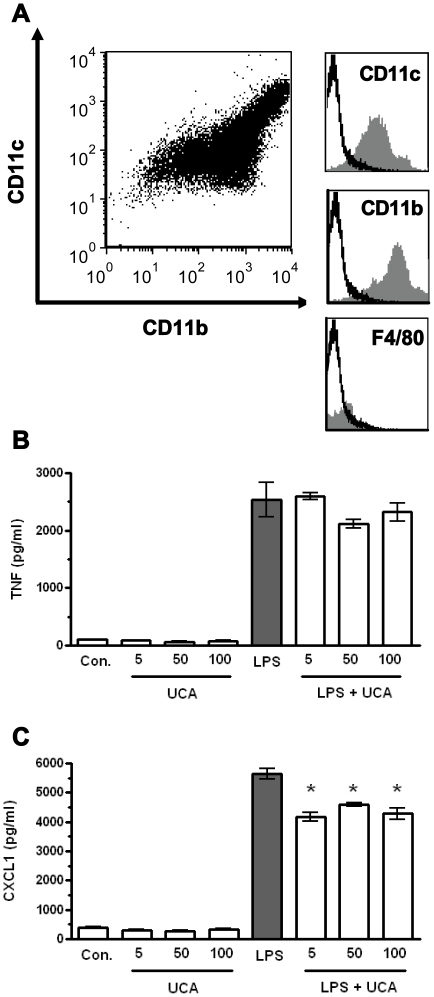
Effect of *cis*-UCA on lipopolysaccharide (LPS)-induced cytokine secretion by bone-marrow-derived antigen presenting cells (BM-APC). BM-APCs derived from long-bones of 129 Sv/EV mice (1×10^6^ cells/ml) were pre-treated for 2 hrs with *cis*-UCA (5 µg, 50 µg, or 100 µg) and then stimulated with LPS (1 µg/ml). Supernatants were collected after 24 hrs. Unstimulated BM-APCs were CD11b+, CD11c+ and F4/80− (A). LPS induced TNFα secretion from BM-APCs and this was not altered by the presence of *cis*-UCA (B). LPS also induced CXCL1 secretion by BM-APCs and this secretion was attenuated by *cis-*UCA (C). n = 4–13 for all measurements. *: p<0.01 compared with LPS.

To determine if the decreased levels of CXCL1 seen in the colonic tissue were due to a direct effect of *cis-*UCA on antigen-presenting cells we examined if *cis-*UCA would modulate cytokine responses to LPS in bone-marrow derived antigen-presenting cells (BM-APCs). BM-APCs were incubated with *cis-*UCA for 24 hrs and TNFα and CXCL1 secretion were measured. As seen in Figure 4B, *cis-*UCA alone did not induce TNFα or CXCL1 secretion. LPS stimulation of BM-APCs cells induced cellular maturation, as evidenced by enhanced TNFα secretion (Fig. 4B). The presence of *cis-*UCA did not alter maturation of BM-APCs cells in response to LPS. However, BM-APCs treated with *cis-*UCA either as concurrent or pre-treatment resulted in a significant (p<0.01) decrease in CXCL1 secretion (Fig. 4C), indicating that *cis-*UCA does have a direct modulating effect on antigen-presenting cell responses to microbial-derived LPS. This response was however not dose dependent at these concentrations, as a significant reduction of CXCL1 secretion was observed for all three doses of *cis-*UCA tested.

### Effect of *cis-*UCA on chronic intestinal inflammation in the IL-10^−/−^ mouse

**Figure 5:**
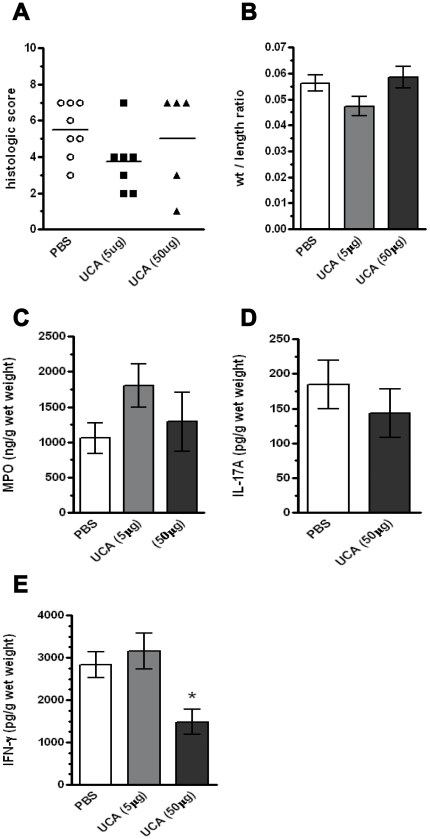
*cis*-UCA does not ameliorate colitis in IL-10^−/−^ mice. 129Sv/EV IL-10^−/−^ mice were injected subcutaneously along the dorsal axis every 2 days for 24 days with either 5 or 50 µg *cis*-UCA, or vehicle (PBS) (n = 5–8). Mice were weighed every second day. *cis*-UCA had no effect on colonic histological score (A), weight/length ratio (B), MPO levels (C), or IL-17A levels (D). *cis*-UCA (50 µg) did reduce levels of colonic IFNγ in IL-10^−/−^ mice as compared with PBS or *cis*-UCA (5 µg) treated (E). *: p<0.05 UCA (50 µg) compared with PBS or UCA (5 µg).

Finally, due to the ability of *cis-*UCA to increase IL-10 secretion [18], coupled with our observation of a trend towards a *cis-*UCA-induced increase in FoxP3+IL-10+ T cells in the DSS studies, we next carried out a series of experiments in the IL-10^−/−^ mouse model of colitis. This mouse model of colitis differs from the DSS-induced acute model, in that a patchy, transmural colitis develops slowly over a period of weeks [22]. We hypothesized that if IL-10 was required for a *cis-*UCA-induced attenuation of gut inflammation, we would see no effect with *cis-*UCA treatment in this model. IL-10^−/−^ mice were treated by subcutaneous injection along the dorsal axis with *cis-*UCA (5 ug or 50 ug) for 24 days. Control IL-10^−/−^ mice received vehicle. As seen in Figure 5, *cis-*UCA at either the high or low dose had no significant effect on disease activity in IL-10^−/−^ mice in either histological score (Fig. 5A) or colon weight to length ratios (Fig. 5B). Furthermore, in contrast to the effects seen in wild-type mice, *cis-*UCA treatment had no effect on colonic tissue homogenate MPO or IL-17A levels (Fig. 5C,D). However, treatment with the high dose of *cis-*UCA did significantly decrease colonic tissue IFN-γ levels compared to treatment with PBS control and low dose *cis-*UCA during colitis in IL-10^−/−^ mice, suggesting a *cis-*UCA-induced modulation of T cell function, even in the absence of IL-10 (Fig. 4E).

## Discussion

This study is the first to examine the effects of *ci*s-urocanic administration in animal models of intestinal inflammation. Here we demonstrate for the first time that subcutaneous injection of *cis-*UCA along the dorsal axis can have several effects on the intestinal inflammatory response during colitis and can attenuate acute colonic disease induced by dextran sodium sulfate. In this study, reduced severity of disease after *cis-*UCA treatment was associated with a decrease in the neutrophil chemoattractant CXCL1, and increased IL-17A in the colon.

Colitis in the DSS model has been shown to be initiated by the innate immune system since disease development can occur in the absence of T and B cells [24]. In this model, oral administration of DSS induces a breakdown in the intestinal epithelial barrier resulting in contact of the luminal microflora with the network of immune cells residing in the lamina propria [24]. In our study, treatment of mice with *cis-*UCA resulted in attenuation of disease severity, as evidenced by reduced disease activity score, weight loss and maintenance of colonic weight/length ratio. This attenuated disease was associated with a decrease in the neutrophil chemoattractant CXCL1. Increased neutrophil infiltration into the crypts and lamina propria with subsequent formation of crypt abscesses in the bowel wall is one of the hallmarks of ulcerative colitis [2], [25]. However the exact contribution of neutrophils to disease pathology in IBD as well as animal models of colitis remains unresolved. Using mice deficient in CXCL1, Shea-Donohue *et al.* demonstrated that a lack of neutrophil infiltration was accompanied by more severe disease compared to wild type mice [26], while Buanne *et al.* demonstrated that mice deficient for the receptor for CXCL1 had reduced neutrophil infiltration and reduced clinical disease in the DSS model [27]. Our findings of reduced disease associated with decreased neutrophilic chemoattractant levels in *cis-*UCA treated mice are consistent with the findings of Buanne et al [27]. *cis-*UCA has been shown to directly alter neutrophilic function by inhibiting the respiratory burst and generation of extracellular superoxide while maintaining the ability of neutrophils to generate intracellular superoxide or other reactive-oxygen species, thus maintaining phagocytic and microbicidal characteristics intact [28]. This would have the effect of limiting bystander tissue injury due to neutrophilic release of reactive oxygen species while maintaining host defence through bacterial clearance.

CXCL1 is produced primarily from macrophages, dendritic cells, and epithelial cells in the gastrointestinal tract in response to microbial stimuli. Our findings that *cis-*UCA treatment had an effect on reducing colonic CXCL1 levels in the presence of DSS could be attributable either to a direct effect of *cis-*UCA on specific cell types, or alternatively, an effect of UCA on colonic barrier function, and a resultant lack of stimulation of resident intestinal immune cells by colonic microflora. Our *in vitro* data supports the concept that *cis-*UCA directly inhibits an LPS-induced CXCL1 secretion from antigen-presenting, but not from epithelial cells. We carried out a series of experiments in HT-29 cultured colonic epithelial cells. HT-29 epithelial cells were pre-treated with various doses of *cis-*UCA and then stimulated with TNFα or LPS. Stimulation of HT-29 cells with *cis-*UCA alone for 3 hrs did not alter IL-8 secretion and had no effect on IL-8 secretion in response to TNFα or LPS (data not shown), suggesting the observed effects *in vivo* were not a direct effect of *cis-*UCA suppressing chemokine secretion from enterocytes. However, as we did not measure the effects of *cis-*UCA on colonic permeability or bacterial translocation in DSS treated mice, we cannot exclude the possibility that *cis-*UCA also had an indirect effect on CXCL1 secretion by preventing interactions between gut microbes and lamina propria immune cells.

An interesting finding in this study was the increased levels of colonic IL-17A in mice treated with *cis-*UCA. IL-17A is secreted by numerous cell types, including Th17 cells, γδ T cells, NK cells, and neutrophils [23], [29]. IL-17A has a major role in chronic autoimmune inflammatory diseases, as well as in the innate immune response to bacterial pathogens [23]. In response to bacterial infection, the primary cell type that secretes IL-17 *in vivo* appears to be the γδ T cell subset that is found predominantly in the gut mucosa [30]. Our finding of increased levels of IL-17A in gut mucosa, with no effect of Th17 cells in the spleen, suggests that *cis-*UCA may be mediating its effects through the γδ T cell subset. Recent studies have demonstrated evidence for a direct role of IL-17-producing γδ T cells, rather than Th17 cells, in the acute defence against pathogens [31]. Other studies have shown that IL-17A produced by γδ T cells establishes an amplification loop by acting directly on Th17 cells to induce their IL-17 production and also indirectly on dendritic cells to increase their IL-23 production [32]. Our data supports these findings, in that increased levels of IL-23 were observed in the *cis-*UCA-treated mice prior to the onset of colitis. A beneficial role for IL-17A in DSS colitis was also demonstrated by Ogawa *et al* [33] who showed that administration of a IL-17 neutralizing antibody during acute DSS colitis resulted in aggravated disease with increased neutrophil infiltration. Further, in the CD45RBhi CD4+ T cell transfer model of colitis, IL-17A mediated protection, rather than inducing inflammation [34]. The ability of IL-17A to protect against bacterial infection and invasion has been linked with its ability to induce antimicrobial peptides and also to increased neutrophil microbicidal activity [35], [36].

Intraepithelial γδT cells act to enhance mucosal protection as well as having an important role in healing of tissue. Murine intestinal γδ+ T cells have been shown to express TGF-β mRNA, secrete IL-10, and inhibit cytotoxic T cell responses [37]. Depletion of γδ+ T cells during acute phases of inflammation has confirmed their protective effect, and has provided evidence for a role for IL-10 secretion from a subset of γδ+T cells in the control of cytotoxic CD8+ T cell expansion [38]. Mice with a deficiency in IL-10 spontaneously develop chronic colitis characterized by infiltration of lymphocytes, monocytes and neutrophils [39]. Disease in these mice is conveyed by Th1 associated cytokines [39]. In this study, administration of *cis-*UCA to IL-10^−/−^ mice did not ameliorate disease, but did significantly decrease colonic IFN-γ levels. *cis-*UCA has been shown to increase IL-10 secretion by CD4+ T-cells *in vitro* and ultraviolet-mediated suppression of contact hypersensitivity and delayed-type hypersensitivity have been shown to be mediated by IL-10 secreting T-regulatory cells [18], [19], [20]. Thus, the lack of efficacy of *cis-*UCA in the IL-10^−/−^ mouse suggests the possibility that IL-10 secreting T-regulatory cells or γδ+ T cells may be critical for *cis-*UCA effects, and further, that the increased levels of IFNγ are not the primary driver of gut inflammation in this model [40].

In conclusion, this study demonstrates that *cis*-urocanic acid is effective in reducing the severity of colitis in a chemically-induced mouse model, indicating that pathways induced by ultraviolet radiation to the skin can influence distal sites of inflammation. This provides further evidence for a possible role for sunlight exposure in modulating autoimmune inflammatory disorders.
